# Multilayer pixel super-resolution lensless in-line holographic microscope with random sample movement

**DOI:** 10.1038/s41598-017-13134-4

**Published:** 2017-10-06

**Authors:** Mingjun Wang, Shaodong Feng, Jigang Wu

**Affiliations:** Biophotonics Laboratory, University of Michigan - Shanghai Jiao Tong University Joint Institute, Shanghai Jiao Tong University, 800 Dong Chuan Road, Shanghai, 200240 China

## Abstract

We report a multilayer lensless in-line holographic microscope (LIHM) with improved imaging resolution by using the pixel super-resolution technique and random sample movement. In our imaging system, a laser beam illuminated the sample and a CMOS imaging sensor located behind the sample recorded the in-line hologram for image reconstruction. During the imaging process, the sample was moved by hand randomly and the in-line holograms were acquired sequentially. Then the sample image was reconstructed from an enhanced-resolution hologram obtained from multiple low-resolution in-line holograms by applying the pixel super-resolution (PSR) technique. We studied the resolution enhancement effects by using the U.S. Air Force (USAF) target as the sample in numerical simulation and experiment. We also showed that multilayer pixel super-resolution images can be obtained by imaging a triple-layer sample made with the filamentous algae on the middle layer and microspheres with diameter of 2 μm on the top and bottom layers. Our pixel super-resolution LIHM provides a compact and low-cost solution for microscopic imaging and is promising for many biomedical applications.

## Introduction

Lensless in-line holographic microscope (LIHM)^1–6^ provides a promising alternative to conventional microscope in application areas such as personal healthcare and telemedicine that requires compact and low-cost microscopic imaging systems. To achieve compactness in LIHM, short sample-to-sensor distance is usually required in the process of recording the holograms. In this case, the resolution of the reconstructed sample image is limited by the finite pixel size of the imaging sensors instead of the numerical aperture of the imaging system. To overcome this resolution limit, researchers have resorted to the pixel super-resolution (PSR) technique which was widely used to improve imaging resolution in photography^7^, holography^8^ and microscopy^9–11^. In LIHM, PSR technique has been successfully applied to reconstruct sub-pixel resolution image from multiple holograms acquired with different light illumination angles^9,10^. In the reported method, a high-resolution hologram was first obtained from multiple low-resolution holograms by the PSR technique, then the high-resolution sample image can be reconstructed from the high-resolution holograms. Nevertheless, this technique requires multiple illumination sources and corresponding control circuits which increase the complexity of the system. This issue can be resolved by using the PSR technique with shifting samples instead of changing the illumination angle. PSR imaging technique with shifting samples has been reported in shadow imaging microscopic techniques^11–13^ and also the holographic opto-fluidic microscopy (HOM)^14^, which is a variation of the LIHM. In the shadow imaging technique, the sample must be attached to or very close to the sensor so as to maintain the microscopic resolution, which limited its applications. In contrast, the HOM solved this problem by using holographic reconstruction. Recently, resolution-enhanced techniques using wavelength scanning was also reported^15,16^ with the additional requirement of a tunable light source or several light sources with different wavelengths.

In the HOM^14^, we notice that the PSR is achieved with sample movement in the microfluidic channel, so the wide field-of-view and multilayer imaging capability of the LIHM is not fully utilized. In this paper, we propose to apply the PSR technique with shifting samples in LIHM for wide field-of-view and multilayer imaging. In our PSR-LIHM system, the shifting sample on a microscope slide is illuminated by a laser beam and the in-line holograms are recorded by an imaging sensor located behind the sample. Similar as previously reported techniques, a high-resolution hologram was reconstructed by applying the PSR technique to multiple low-resolution holograms. Then a high-resolution sample image was reconstructed from the high-resolution hologram. With our imaging method, on the one hand, only one illumination source is needed which reduces the system complexity and cost. On the other hand, thanks to the holographic reconstruction principle, the sample can be detached from the imaging sensor and the sample-to-sensor distance is not critical compared to the shadow imaging case. Furthermore, the movement of the sample is driven by hand randomly in our experiment without any moving stages or motion control devices, which greatly simplifies the imaging system. We built a prototype system to demonstrate the capability of our technique in acquiring microscopic PSR images of the U.S. air force (USAF) target and a triple-layer biological samples consisting of filamentous algae and microsphere with a field of view of 5.7 * 4.3 mm.

## Principles

**Figure 1:**
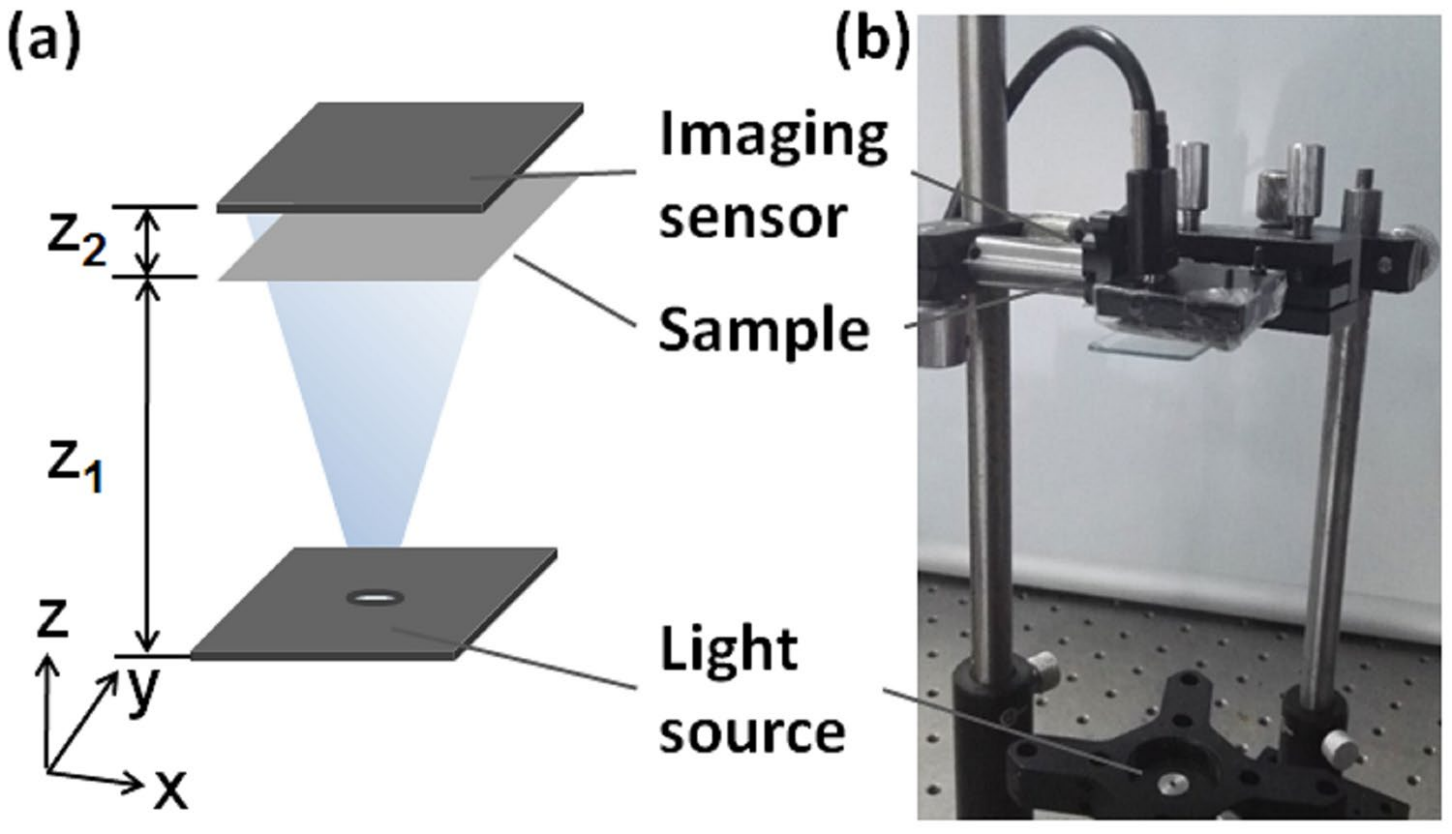
(**a**) The schematic diagram of our imaging system setup, where the sample can have multilayer structure. z_1_ was ~8 cm, and z_2_ was 1~3 mm in our experiment; (**b**) The photograph of the experimental system.

The schematic diagram and the photograph of the imaging system setup are shown in Fig. 1 and explained in the Methods section. As shown in the figure, our imaging system setup is very simple and can be easily integrated as a compact imaging system.

**Figure 2:**
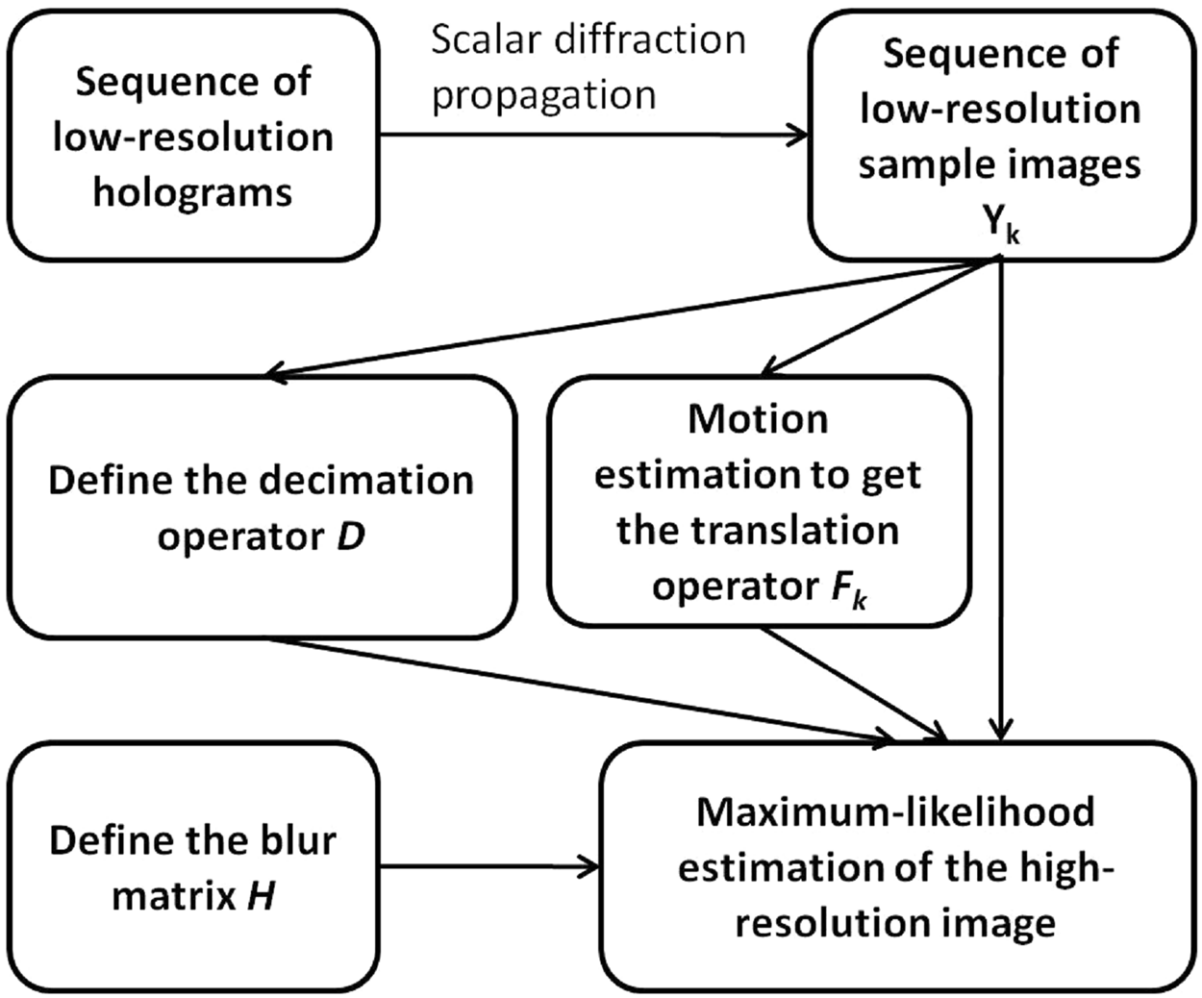
The flow chart of reconstructing images with enhanced-resolution in our PSR-LIHM imaging system.

The principle of reconstructing images with enhanced-resolution in our PSR-LIHM imaging system is illustrated in the flow chart as shown in Fig. 2. During the imaging process, a sequence of low-resolution holograms was captured by the CMOS imaging sensor while the sample was randomly and slowly shifted by hand. Then the holograms were reconstructed to get the low-resolution sample images before applying the PSR technique to get a high-resolution sample image. We used the angular spectrum propagation method in scalar diffraction^17^ for the holographic reconstruction by assuming plane wave illumination. For multilayer sample, different holographic reconstruction distances needed to be used for different layers. In the PSR reconstruction, we used the fast noniterative algorithm reported by M. Elad *et al*.^18–20^ with the assumption of pure translational motion and common space-invariant blur. In this algorithm, the low-resolution images Yk (k = 1, …, N) are modeled as

1 $${Y}_{k}=DH{F}_{k}X+{V}_{k},\quad \quad k=1,\mathrm{...},N$$

where X is the high-resolution image, F_k_ is the translation operator, H is the blur matrix representing the imaging point spread function, D is the decimation operation representing the loss of resolution in obtained images, and V_k_ is the additive measurement noise with auto-correlation matrix E{V_k_V_k_^T^} = W_k_.

In our experiment, the sample was moved by hand randomly, and F_k_ was estimated through an image registration process, where the cross-correlation of the low-resolution images was calculated and the relative shifts between them were determined by the maximum cross-correlation value. The blur matrix H was assumed to be a circular disk and the radius was chosen in the final image deblur process to optimize the image quality. The decimation operator D was defined according to the pixel enhancements in the high-resolution image, where we used 4 * 4 pixel enhancements in our simulations and experiments. The feasibility of PSR with random shifting was already demonstrated in other literatures^14,21^. The maximum likelihood estimation of X can then be written as

2 $$\hat{X}=\mathop{{\rm{\arg }}{\rm{\min }}}\limits_{X}\{\sum _{k=1}^{N}{[{Y}_{k}-DH{F}_{k}X]}^{T}{W}_{k}^{-1}[{Y}_{k}-DH{F}_{k}X]\}$$

and $\hat{X}$ can be computed noniteratively as

3 $$\hat{X}={H}^{-1}{\tilde{R}}^{-1}\tilde{P}$$

where

4 $${\tilde{R}}^{-1}=\sum _{k=1}^{N}{F}_{k}^{T}{D}^{T}D{F}_{k},\,\tilde{P}=\sum _{k=1}^{N}{F}_{k}^{T}{D}^{T}{Y}_{k}$$

## Results

During the experiment, we observed that the movement of the sample should be smooth enough to adapt for the exposure time of the imaging sensor, and the moving range of the sample should also be limited for better PSR reconstruction. To control the sample movement, in our prototype system, the sample was loosely mounted on a simple mechanical mount (GCM-1301M, Daheng New Epoch Technology Inc., China) and moved with hand carefully and randomly.

**Figure 3:**
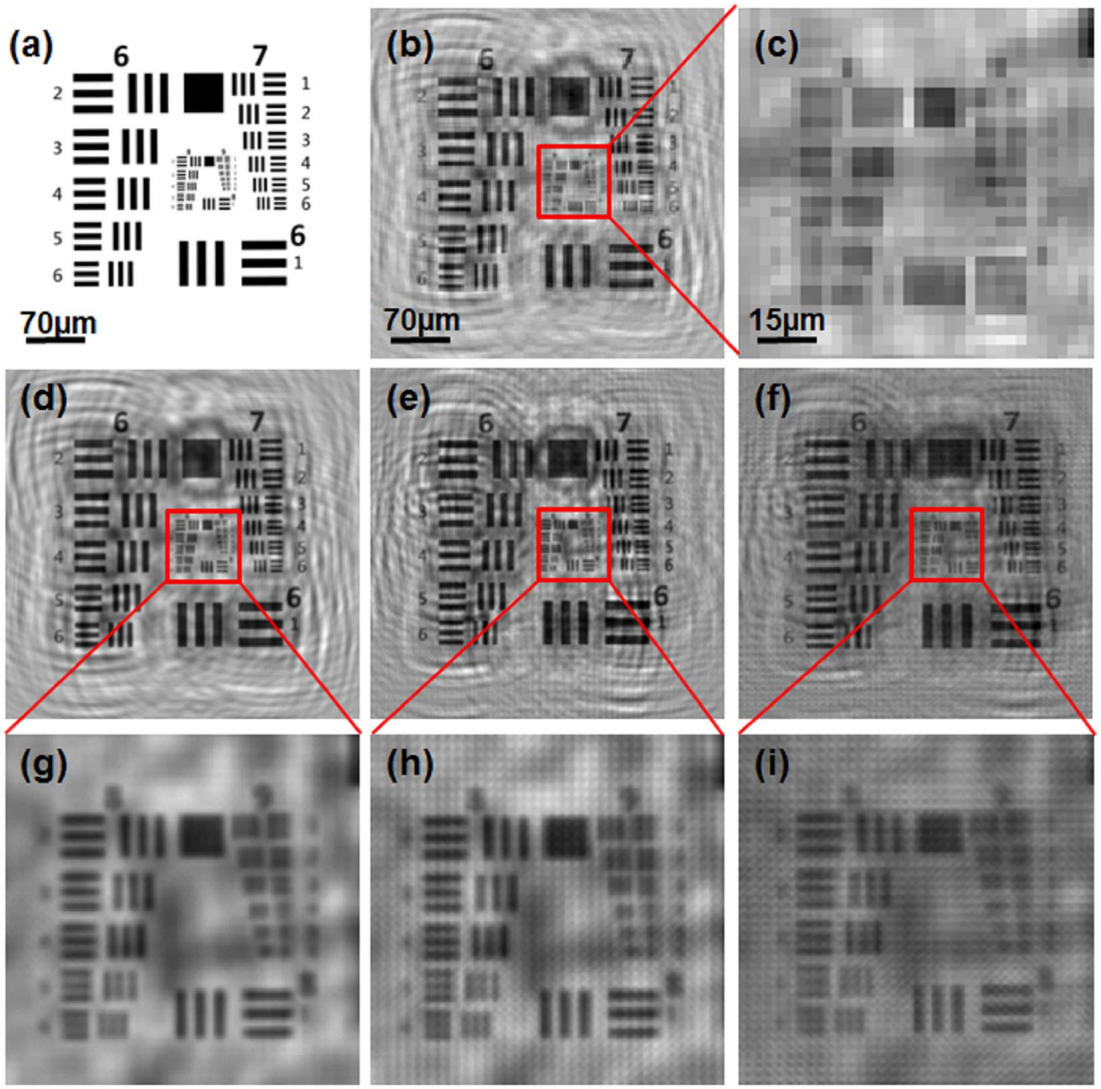
Numerical simulation to study the performance of the PSR-LIHM system. (**a**) original testing target; (**b**) holographic reconstruction of the target using a low-resolution hologram; (**c**) enlarged image of the region indicated in (**b**); (**d**,**e**,**f**) high-resolution target image reconstructed with the PSR algorithm for maximum sample moving range of 22 μm, 66 μm, and 110 μm, respectively; (**g**)(**h**)(**i**) enlarged images of the regions indicated in (**d**,**e**,**f**), respectively.

To study the performance of our PSR-LIHM system and the effect of sample moving range, we did numerical simulations with an ideal USAF target image using the same experiment configuration parameters as described in previous section. Figure 3(a) shows the simulated USAF target image. During the simulation, the original target image pixel size was set as 0.22 μm and the in-line hologram in the sensor plane was obtained by numerically propagate the target image by scalar propagation. Then 50 low-resolution holograms with pixel size of 2.2 μm were generated by random shifting in xy-plane with uniform distribution and then down-sampling. These low-resolution holograms were then used to reconstruct the high-resolution sample image with 4 * 4 pixel enhancement in pixels using the PSR technique. Figure 3(b) shows the holographic reconstruction of the target using one of the low-resolution hologram and Fig. 3(c) shows the enlarged region indicated in Fig. 3(b). We can see that the group 8 and 9 of the USAF target (line width of all the elements <2 μm) cannot be resolved because the insufficient image pixel size of 2.2 μm. Figure 3(d,e,f) show the high-resolution target image reconstructed with the PSR algorithm for maximum sample moving range of 22 μm, 66 μm, and 110 μm, respectively and Fig. 3(g,h,i) show the enlarged images of the regions indicated in (d)(e)(f), respectively. We can clearly see that the element 6 of group 8 with line width of 1.1 μm can be resolved for small moving ranges, and there will be reconstruction artifact for large moving ranges.

**Figure 4:**
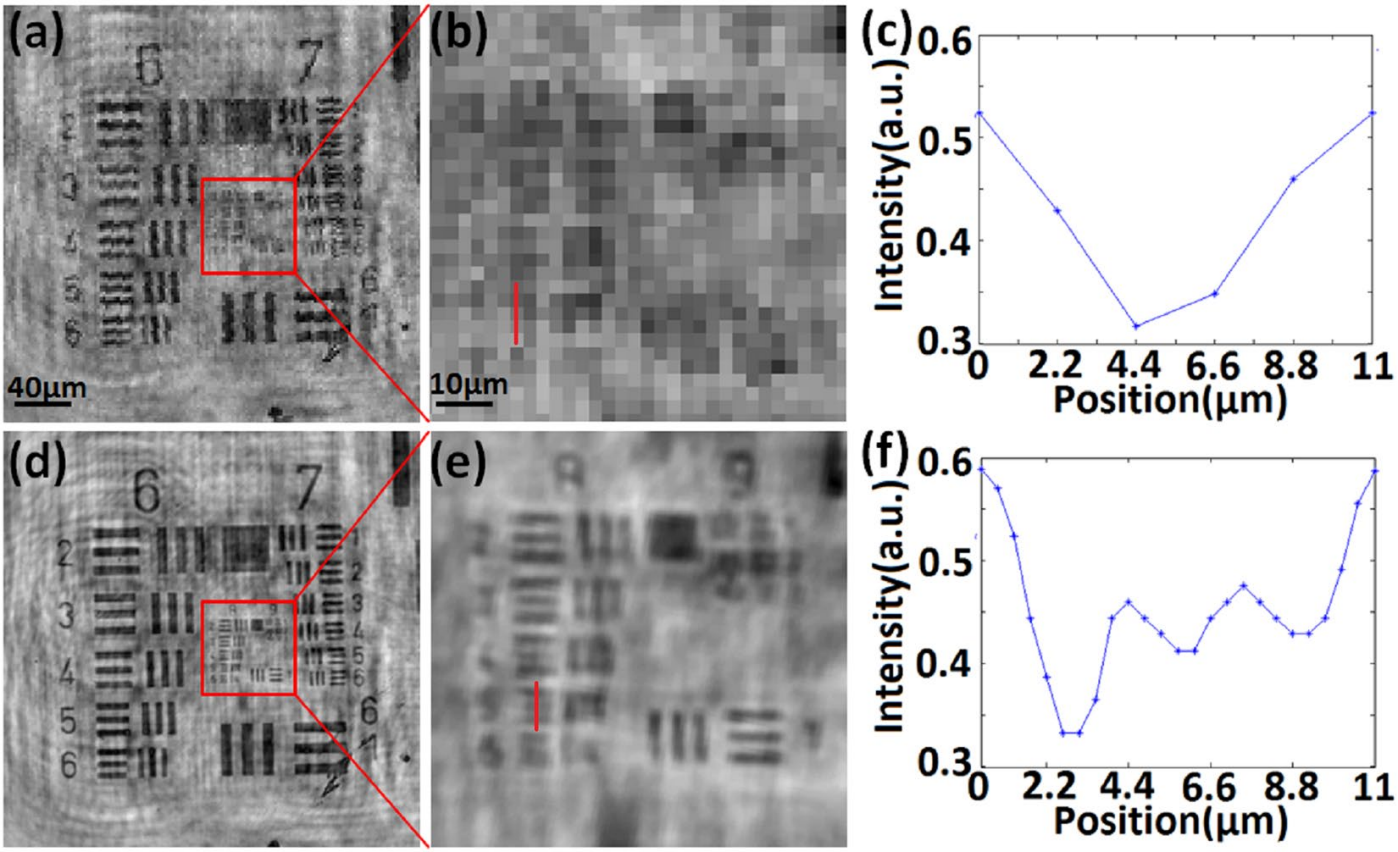
Experimental demonstration of the PSR-LIHM system by imaging the USAF target. (**a**) holographic reconstruction of the target using a low-resolution hologram; (**b**) enlarged image of the region indicated in (**a**); (**c**) cross-sectional profile of element 5, group 8 as indicated in (**b**); (**d**) high-resolution target image reconstructed with the PSR algorithm; (**e**) enlarged image of the region indicated in (**d**); (**f**) cross-sectional profile of element 5, group 8 as indicated in (**e**).

Next, we applied the PSR-LIHM system to image the USAF target experimentally. During the experiment, the USAF target was randomly moved with hand, and 50 in-line holograms were recorded by the CMOS imaging sensor with a frame rate of 52 fps and exposure time of 2.85 ms. The moving speed was estimated to be around 20–30 μm/sec and the reconstruction distance was 1380 μm. Figure 4(a) shows the low-resolution sample image holographically reconstructed from one of the in-line holograms and Fig. 4(b) shows the enlarged image of the region indicated in Fig. 4(a). As expected, the group 8 and 9 of the USAF target cannot be resolved. Figure 4(c) shows the cross-sectional profile of element 5, group 8 (line width of 1.23 μm) as indicated in Fig. 4(b). In contrast, Fig. 4(d) shows the high-resolution target image obtained with PSR algorithm mentioned previously, and Fig. 4(e) shows the enlarged region indicated in Fig. 4(d). We can see that the USAF target can be better resolved, and the cross-sectional profile of element 5, group 8 is shown in Fig. 4(f).

We mentioned that the PSR-LIHM system can be used for multilayer sample imaging. For a preliminary demonstration, we made a triple-layer sample for the imaging experiment. The triple-layer sample consisted of the filamentous algae on the middle layer and microspheres with diameter of 2 μm on the top and bottom layers. The middle layer is separated from the bottom layer with a glass slide of thickness ~1100 μm and from the top layer with a cover glass of thickness ~260 μm. Similar as previous experiment, we obtained 50 low-resolution holograms while moving the sample randomly in order to reconstruct the high-resolution image with the PSR algorithm. The sample images at the three layer positions were reconstructed with distances of 1940 μm, 2100 μm and 2855 μm, respectively, where the distances were found by the Tenenbaum gradient auto-focusing algorithm^22^. Notice that the differences in the reconstruction distances are 160 μm and 755 μm, consistent with the thicknesses of the glass slide and the cover glass considering the glass refractive index of ~1.5.

**Figure 5:**
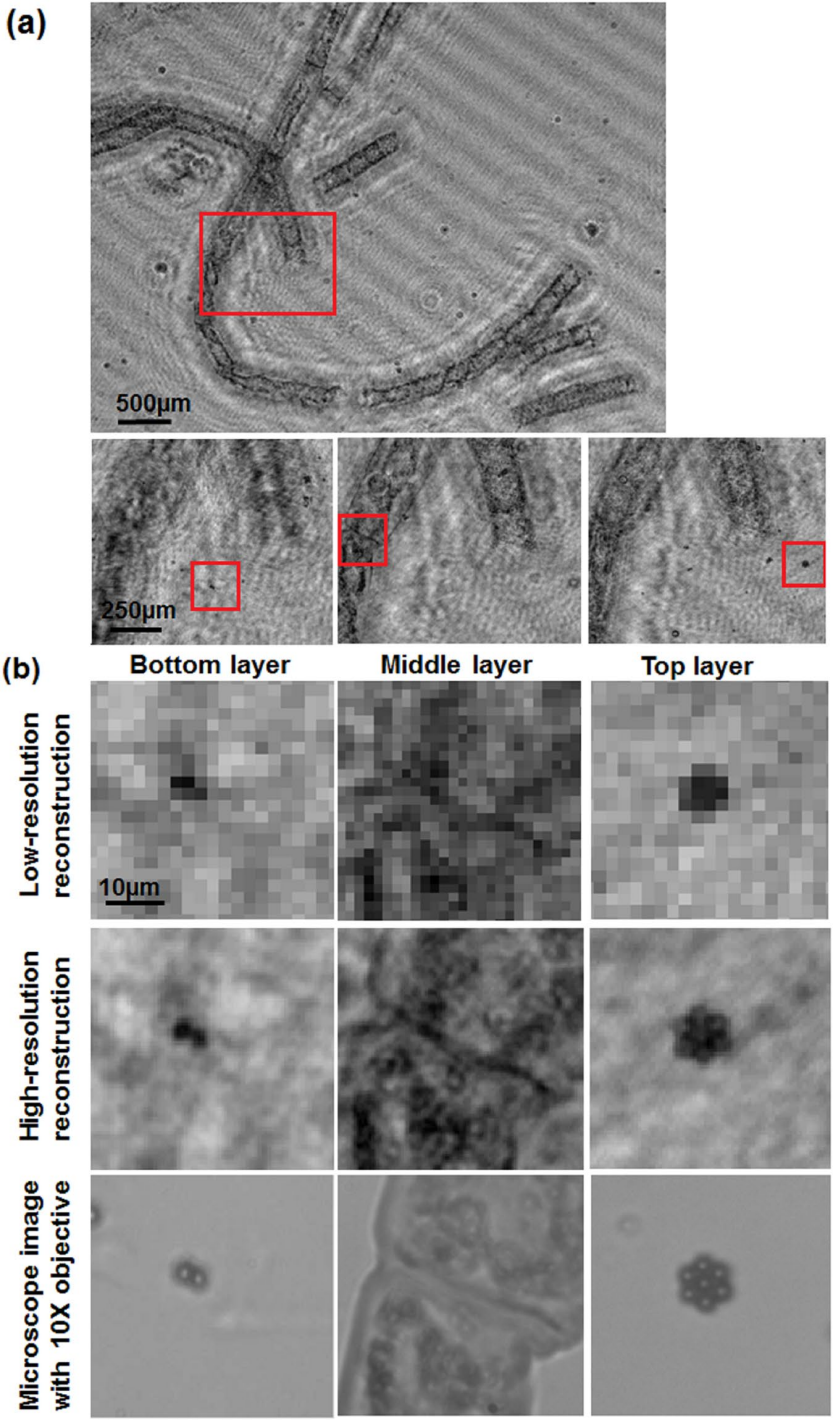
(**a**) Full field-of-view (5.7 * 4.3 mm) image and the enlarged images reconstructed at the three layer positions of the triple-layer sample made with filamentous algae in middle layer and the 2 μm-diameter microspheres in the bottom and top layers; (**b**) enlarged images of the regions indicated in (**a**). The three rows indicate the low-resolution images, high-resolution images and the microscope images with 10X objective of the three layers respectively.

Figure 5(a) shows the full field-of-view reconstruction image of the triple-layer sample and the enlarged images reconstructed at the three layer positions, where the field of view is the same as the imaging sensor size, i.e., 5.7 * 4.3 mm, and the three enlarged images show different focusing planes. Figure 5(b) shows the enlarged images of regions indicated in the square boxes of the bottom, middle and top layers in Fig. 5(a) at reconstruction distances of 1940 μm, 2100 μm and 2855 μm, respectively. The top row shows the low-resolution images with direct holographic reconstruction. The middle row shows the high-resolution images with PSR algorithm using 50 low-resolution images. And for comparison, the bottom row shows the microscope images with 10X objective. Again, we can obviously see the resolution enhancement by applying the PSR algorithm.

**Figure 6:**
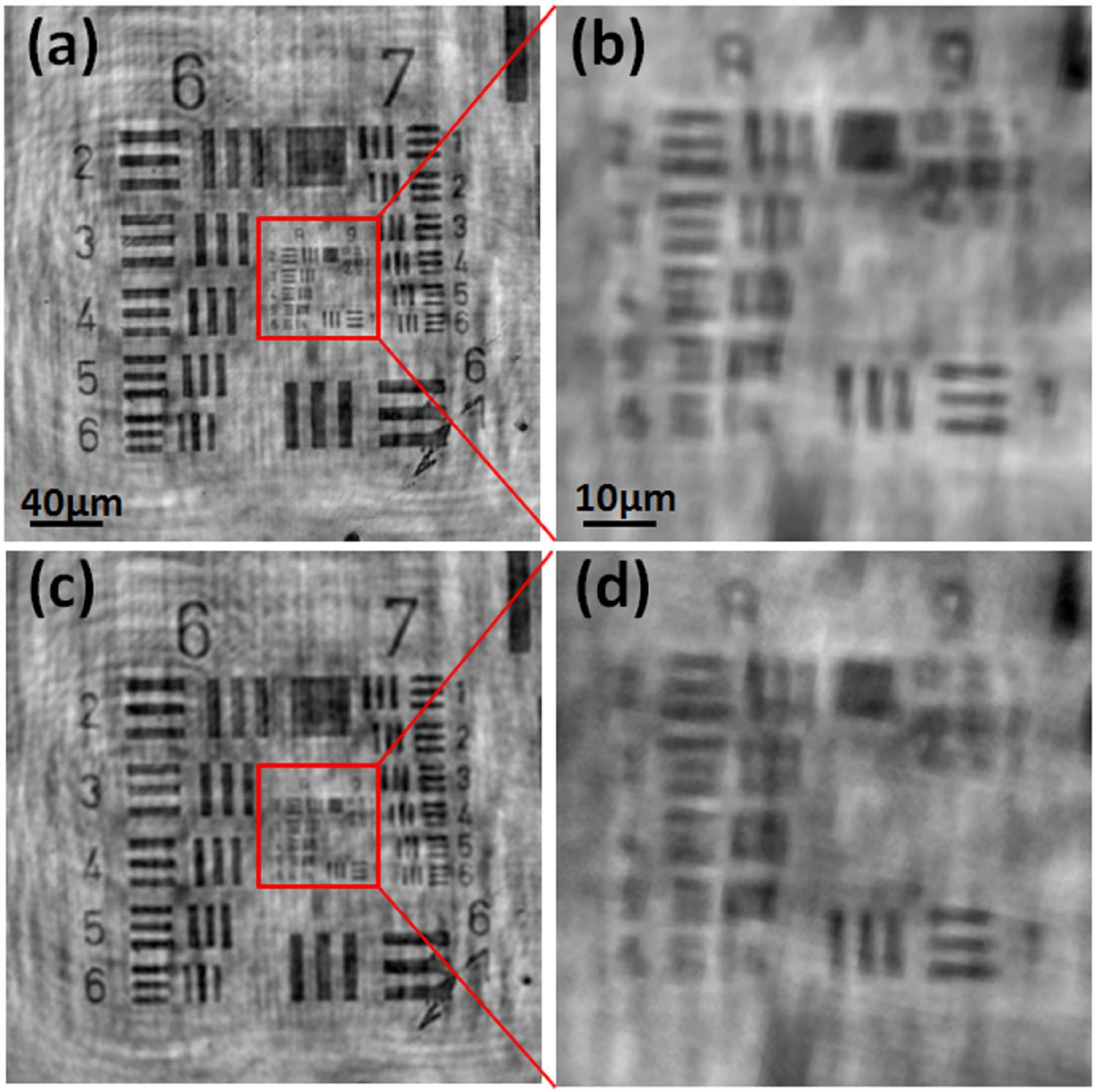
(**a**) PSR-LIHM image of the USAF target with moving ranges of 22 μm; (**b**) PSR-LIHM image of the USAF target with moving ranges of 85 μm.

To study the effect of moving range experimentally, we also performed imaging experiment of the USAF target with larger moving ranges. Figure 6(a,b) is a copy of Fig. 4(d,e), which is the image of the USAF target reconstructed with the PSR algorithm with moving range of 22 μm, and Fig. 6(c) is the image of the USAF target reconstructed with the PSR algorithm with moving range of 85 μm. We can see that larger moving range results in worse PSR reconstruction as expected.

## Discussion and Conclusion

Since we used standard in-line holographic reconstruction in our imaging system, the lateral and axial resolution of the low-resolution reconstructed sample images is similar as in conventional in-line holography^2^. In our method, we showed that the PSR technique can enhance the lateral imaging resolution and the enhancement depends on the frame number of low-resolution images and the estimation precision of the relative shifts. In our experiment, the total thickness of our multilayer sample was much smaller than the distance between the sample and the sensor, thus the imaging resolution and quality for different layers is similar. However, if the thickness of multilayer sample is significant compared to the sample-to-sensor distance, the lateral resolution will be different for different layers, where the imaging resolution will be limited by the imaging numerical aperture, thus the sensor size, for remote sample, and by the sensor pixel size for short sample-to-sensor distance.

Notice that our current holographic reconstruction will result in the twin-image background which can be observed in our imaging results. Furthermore, other background noise arising from scattering of different part of the system, especially for thick samples, will also affect the PSR reconstruction. Further study is required to improve the imaging performance by getting rid of these background noises.

In conclusion, we reported a pixel super-resolution lensless in-line holographic microscope (PSR-LIHM) with random sample movement and demonstrated its capability in resolution enhancement by imaging the USAF target and biological samples. We obtained the enhanced-resolution images at three different layers simultaneously of a triple-layer sample with a field of view of 5.7 * 4.3 mm. We also discussed the effect of moving range and show that satisfactory images can be obtained with a small moving range of 22 μm using our current experimental parameters. Because of its simple imaging setup, the PSR-LIHM system is low cost and can be easily integrated into compact devices, and is thus promising for many biological imaging applications.

## Methods

As shown in Fig. 1, the sample was illuminated by a fiber-coupled output from a laser with wavelength of 473 nm (MBL-III-473, Changchun New Industries Optoelectronics Technology Co., China). The output light power from the fiber tip was 8 mW. The distance between the laser output fiber tip and the sample plane was ~8 cm. Here the sample can have multilayer structure with different z positions. A CMOS imaging sensor with 2592 * 1944 pixels and pixel size of 2.2 µm (DMK72AUC02, the Imaging Sources Europe GmbH) was placed behind the sample to record the in-line holograms formed by the interference of the scattered and unscattered light at the sensor plane. The distance between the sample plane and the sensor plane was ~1–3 mm. Notice that compared to the short sample-to-sensor distance and the field-of-view of the imaging system (5.7 * 4.3 mm), the large distance between the fiber tip and the sample plane ensured that the illumination light on the sample plane can be approximately assumed as plane wave during the holographic reconstruction.
